# Differential virulence gene carriage in *Listeria monocytogenes* strains from neonatal sepsis: insights from whole-genome sequencing of a multicenter cohort

**DOI:** 10.3389/fmicb.2026.1869258

**Published:** 2026-07-14

**Authors:** Le Wang, Lingzhi Meng, Zelei Liu, Zhijie Wen, Hongbin Zhu, Wei Guo, Yinghui Guo, Li Ma

**Affiliations:** 1 Institute of Pediatric Research, Children’s Hospital of Hebei Province, Shijiazhuang, Hebei, China; 2 Department of Neonatology, Children’s Hospital of Hebei Province, Shijiazhuang, Hebei, China; 3 Department of Neonatology, Wu'an First People's Hospital, Wu'an, China; 4 Department of Neonatology, Qinhuangdao Maternal and Child Health Hospital, Qinhuangdao, China; 5 Department of Neonatology, Xingtai People's Hospital, Xingtai, China; 6 Department of Medical Laboratory, Children’s Hospital of Hebei Province, Shijiazhuang, Hebei, China

**Keywords:** comparative genomics, *Listeria monocytogenes*, neonatal sepsis, virulence genes, whole-genome sequencing

## Abstract

**Background:**

Neonatal *Listeria monocytogenes* (*L. monocytogenes*) sepsis is rare but clinically severe. Existing research primarily focused on epidemiology, clinical characteristics, and prognosis, whereas WGS-based descriptions of virulence gene repertoires in neonatal isolates remain limited.

**Methods:**

This multicenter retrospective case series analyzed 11 neonates diagnosed with culture-confirmed *L. monocytogenes* sepsis. Perinatal data, clinical presentations, interventions, complications, and outcomes were recorded. Whole-genome sequencing (WGS) was performed on four available *L. monocytogenes* isolates, with virulence gene profiles annotated against public databases. We summarized the carriage of selected virulence genes, including *inlP, actA, auto, vip,* and *bsh*, and interpreted these profiles together with case-level clinical severity and phylogenetic background.

**Results:**

All cases were early onset, predominantly affecting preterm infants, requiring respiratory support, and with a high incidence of severe complications. All four sequenced isolates harbored core virulence genes associated with adhesion, invasion, motility, and stress survival. The nSOFA scores for Cases 1–4 were 4, 7, 2, and 2, respectively. Case-level differences were observed in the carriage of virulence genes, including *actA*, *auto*, *vip,* etc. Core-genome SNP phylogenetic analysis revealed that the four isolates belonged to different clonal complexes, with Cases 1 and 2 belonging to the CC1/lineage I, and Cases 3 and 4 belonging to the lineage II-related clonal complex, including CC155 and CC121. Expanded analysis incorporating 39 public reference genomes suggested that the selected accessory virulence genes, particularly *actA* and *bsh* carriage, should be interpreted within the MLST lineage background rather than as direct indicators of clinical severity.

**Conclusion:**

This multicenter study provides preliminary clinical and genomic data on neonatal *L. monocytogenes* sepsis. The observed heterogeneity in virulence gene profiles indicates genomic diversity among isolates and should be interpreted within a phylogenetic context. Larger-scale, lineage-matched cohort studies and functional investigations are needed before any genotype–phenotype relationships can be inferred.

## Introduction

*Listeria monocytogenes* (*L. monocytogenes*) is a critical foodborne pathogen capable of causing invasive listeriosis through the ingestion of contaminated food (Jeffs et al., 2020). Pregnancy confers heightened susceptibility, and placental infection may result in adverse pregnancy outcomes as well as early-onset neonatal infection, including sepsis and meningitis (Ke et al., 2022). Neonatal *L. monocytogenes* sepsis is typically early onset and may be complicated by pneumonia, respiratory failure, meningitis, and septic shock. Despite standard treatment, the mortality rate remains substantial (Qu et al., 2022).

In our prior multicenter study across 23 hospitals within the Hebei Provincial Neonatal Sepsis Research Collaboration Group, neonatal *L. monocytogenes* sepsis was characterized by onset within 3 days of birth, frequent prematurity and chorioamnionitis, substantial need for respiratory support (including mechanical ventilation), and a high burden of complications and death (Group HPNSRC, 2025). In the previous work, penicillin-resistance determinants were not observed in the sequenced isolates, yet clinical severity varied markedly across cases. This observation suggests that inter-strain differences in virulence potential—rather than antimicrobial resistance—may contribute to phenotype heterogeneity.

Most neonatal and perinatal *L. monocytogenes* studies have emphasized epidemiology, clinical manifestations, treatment, and outcomes, typically treating invasive *L. monocytogenes* infection as a single exposure without stratification at the isolate level (Charlier et al., 2023; Li et al., 2023). Whole genome sequencing (WGS) has been applied to invasive *L. monocytogenes* cases to investigate genetic features associated with central nervous system infection (Cardenas-Alvarez et al., 2022), vertical transmission (Luo et al., 2019), or systemic diseases (LaTuga, 2025). It has also been used to characterize virulence gene repertoires in food and clinical isolates, together with *in vitro* adhesion and invasion assays (LaTuga, 2025). However, the description of the virulence genes of neonatal *L. monocytogenes* sepsis isolates based on WGS remains limited, especially when interpreted alongside the detailed clinical metadata and phylogenetic background.

This study is a secondary exploratory analysis of a multicenter neonatal *L. monocytogenes* sepsis cohort (Group HPNSRC, 2025). Based on that cohort, we performed WGS-based virulence gene profiling on four available blood-culture isolates and integrated public reference genomes to interpret specific virulence gene carriage patterns within the context of MLST lineages and CCs. We used the nSOFA score to describe clinical severity. Given the small sample size, this analysis is intended to generate testable hypotheses for larger, prospective genotype–phenotype studies in neonatal *L. monocytogenes* sepsis.

## Methods

### Study design and ethics

This multicenter retrospective case series is nested within our collaborative cohort for the Neonatal Sepsis Study. This cohort included newborns diagnosed with *L. monocytogenes* sepsis by blood culture at 23 municipal hospitals in Hebei Province, and their bacterial strains were recorded. The study period was from November 1, 2021, to December 31, 2022. The study was approved by the Ethics Committee of Hebei Provincial Children’s Hospital (Medical research ethics review no. 202103), and ethical approval was also obtained from all collaborating hospitals. That study identified 11 neonatal cases using the same case definitions and data sources as previously reported (Group HPNSRC, 2025). However, this study explored different research questions and incorporated new genomic analyses. Four LM isolates from these 11 cases were available for WGS and virulence gene annotation. Clinical variables are summarized only as needed to interpret the WGS results; detailed clinical data and antimicrobial resistance profiles for all 11 cases have been reported in our previous publications.

### Patients and definitions

According to the Expert Consensus on the Diagnosis and Treatment of Neonatal Sepsis (2019 Edition) (Subspecialty Group of Neonatology tSoPCMA and Professional Committee of Infectious Diseases NSCMDA, 2019), the inclusion criteria were: (1) newborns within 28 days of birth or premature infants with a corrected gestational age less than 44 weeks; (2) presence of infection-related clinical symptoms and a positive LM blood culture. The exclusion criteria were: (1) failure to retain bacterial strain specimens or perform WGS analysis; (2) presence of complex congenital structural malformations; (3) severe lack of clinical or laboratory data that could not be analyzed.

The study used the neonatal sequential organ failure assessment (nSOFA) scoring system to evaluate the clinical severity of neonatal sepsis. The nSOFA scoring system is widely used in neonatal intensive care, primarily quantifying organ dysfunction in newborns based on the functional status of the respiratory, cardiovascular, and blood (platelet) systems (Wynn and Polin, 2020). The time point for taking the value is the maximum value within 24 h after the onset of the disease. Each organ system is scored from 0 to 4 points, with higher scores indicating greater clinical severity.

**Figure 1:**
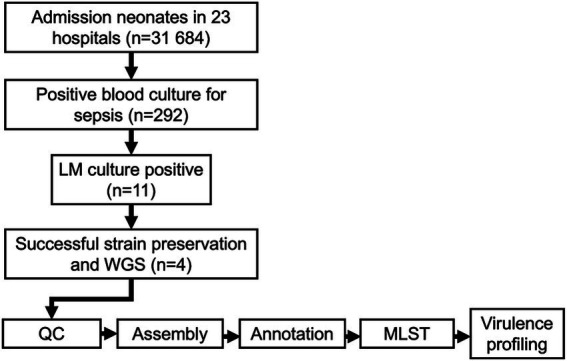
Flowchart of the study and whole-genome sequencing (WGS) analysis pipeline.

This multicenter study included 23 municipal and higher-level medical institutions: 2 children’s hospitals, 6 maternal and child health hospitals, and 15 general hospitals. During the study period, these centers admitted a total of 31,684 newborns. Among them, 292 neonates were diagnosed with early-onset sepsis. They had positive blood cultures, including 11 cases of blood culture-confirmed *L. monocytogenes* sepsis (3.7%). Thus, *L. monocytogenes* accounted for 3.7% of blood culture-positive early-onset neonatal sepsis cases in this multicenter cohort. This proportion represents the pathogen composition among culture-confirmed neonatal sepsis cases, rather than a population-based incidence rate. When calculated using all hospitalized neonates as the denominator, the detection proportion of neonatal *L. monocytogenes* sepsis was 0.34 per 1,000 hospitalized neonates (Group HPNSRC, 2025). Of the 11 cases confirmed by blood culture, 4 clinical isolates were successfully preserved, resuscitated, and usable for WGS. The remaining 7 isolates could not be included in further analysis due to the original strain not being preserved, insufficient preservation conditions for subsequent resuscitation, or failed resuscitation after subculturing. Therefore, the usability of isolates for sequencing depends on the feasibility of preservation and resuscitation of routine clinical strains, rather than clinical severity or prognostic selection (Figure 1).

### Clinical data collection

Each collaborative center uses a pre-designed electronic case report form (CRF), with data prospectively entered by trained, dedicated researchers. The data included:

1. *Perinatal data*: sex, gestational age, birth weight, mode of delivery (vaginal/cesarean section), multiple pregnancy; maternal pregnancy complications (chorioamnionitis, gestational hypertension, gestational diabetes, etc.). Diagnosis of chorioamnionitis was based on international consensus and placental pathology results.
2. *Clinical presentation and treatment*: onset time, main symptoms, and signs; respiratory support methods and maximum support level; empirical antimicrobial therapy and definitive antimicrobial therapy after obtaining etiological results.
3. *Complications and outcomes*: occurrence of purulent meningitis, intraventricular hemorrhage and/or cerebral hemorrhage, septic shock, disseminated intravascular coagulation (DIC), etc.; length of hospital stay, death, or withdrawal of care.
4. *Laboratory tests*: the first complete blood count (white blood cell count, percentage of neutrophils, platelet count), C-reactive protein (CRP), procalcitonin (PCT) and other tests were performed within 24 h after admission.

### Microbiology and WGS

Genomic DNA was extracted from pure cultures and assessed for concentration and integrity (NanoDrop; agarose gel electrophoresis). Libraries were prepared using the Hieff^®^ NGS OnePot Pro DNA Library Preparation Kit V2 (Yeasen Biotechnology), which facilitates enzymatic fragmentation, end-repair, and adapter ligation in a single reaction. Sequencing was performed on the Cygnus Biosciences S100 sequencing platform (PE150 mode). The raw sequencing data were first preprocessed using FASTP software (v0.23.4) for comprehensive quality control and filtering, removing reads with low overall or terminal base quality values and reads containing adapter sequences, thus obtaining high-quality reads for subsequent analysis (Chen, 2023). Subsequently, high-quality reads were *de novo* assembled using SPAdes (v3.15.3) to obtain scaffold/contig sequences (Prjibelski et al., 2020). The open reading frame (ORF) prediction was performed using Prodigal (v2.6.3) to obtain coding gene sequence information (Hyatt et al., 2010). Additionally, the multi-locus sequence typing (MLST) Sequence Types (STs) and Clonal Complexes (CCs) of the four clinical isolates were determined in silico for downstream phylogenetic context mapping.

### Virulence factor annotation

To annotate virulence factors, we employed two alignment strategies: DIAMOND blastx (v2.0.14.152) and hs-blastn (v0.0.5) (Chen et al., 2015). The former aligns predicted gene translation sequences with the protein database of the Virulence Factor Database (VFDB), while the latter performs DNA–DNA alignment of contig sequences with the VFDB nucleic acid database to identify potential virulence factors (Chen et al., 2015; Buchfink et al., 2021). Both alignment methods used uniform thresholds: minimum coverage ≥ 60% and minimum sequence identity ≥ 80%. We used coverage and sequence identity thresholds to screen candidate genes to minimize false positives from short-fragment matches and avoid accidentally deleting variant-containing genes (Zankari et al., 2012). Finally, based on the threshold screening results, we compiled the virulence gene profile for each strain, focusing on typical virulence factors associated with adhesion, invasion, intracellular motility, and stress survival.

### Reference genome selection and core genome SNP phylogeny

To accurately elucidate the evolutionary position and phylogenetic background of our new isolates, a rigorous multi-step screening strategy was implemented, selecting globally representative reference genomes from a well-validated consensus dataset.

1. *Initial database screening*: based on the comprehensive international dataset published by Moura et al. (2016), which served as the baseline genome database for comparison, this dataset contained 1,696 representative *L. monocytogenes* strains.
2. *Quality control and single-strain validation*: to ensure maximum sequence fidelity for subsequent variant detection, we filtered out redundant or unassembled database entries in this dataset. We systematically excluded BioProject records associated with multiple or non-unique genome assemblies. Furthermore, we removed raw short read sequences (e.g., SRR/ERR/DRR accession numbers) and raw sample descriptors lacking fully validated assembly reference profiles, ultimately retaining a subset of 1,584 high-quality genome entries after screening.
3. *Non-redundant stratification by clonal complex*: to prevent phylogenetic tree skew caused by local overexpression of specific common clinical or environmental clusters, these high-quality reference genomes are stratified according to their MLST and CCs.
4. *Representative strain selection*: within each CC group, genomes are prioritized based on metadata richness. Specifically, the genome with the most recent isolation year and complete geographic metadata (including explicit country information) is selected as the sole non-redundant representative of that particular clonal complex.

Through this deterministic curation process, a robust reference panel of 39 representative *L. monocytogenes* genomes was established, comprehensively covering Pathogenic Lineage I (e.g., hyper-virulent clones CC1, CC2, CC4, and CC6), Lineage II (e.g., environmental/food-associated clones CC7, CC9, CC121, and CC155), and the rare evolutionary branches within Lineage III and Lineage IV. One *Listeria innocua* genome Clip11262, also obtained from the reference framework, was integrated as an outgroup to root the tree.

For variant calling, high-quality paired-end reads of our 4 clinical isolates were aligned against the *L. monocytogenes* reference genome EGD-e (GenBank accession: NC_003210) via the Snippy pipeline, and integrated with the 39 public genomes selected above. A core genome alignment was generated using snippy-core. To ensure the accuracy of the phylogenetic inference, Gubbins was employed to identify and mask chromosomal regions associated with recombination. Subsequently, single-nucleotide polymorphisms (SNPs) were extracted from the filtered alignment using snp-sites (parameter -c), with all non-variant positions excluded. Maximum-likelihood (ML) phylogenetic trees were reconstructed from the core SNP alignment using FastTree under the Generalized Time-Reversible (GTR) model of nucleotide substitution (parameters -gtr -nt). The resulting phylogeny was visualized and annotated using the Interactive Tree Of Life (iTOL) platform (Letunic and Bork, 2019). Isolates were assigned to specific lineages based on their clustering patterns relative to the reference strains. For visualization, the dataset was partitioned into five distinct groups: Lineage I (red), Lineage II (blue), Lineage III (green), Lineage IV (purple), and the outgroup (grey).

### Data analysis

Due to the limited sample size in this study, descriptive statistical methods were primarily employed. Continuous variables were expressed as median and quartiles (M [P25, P75]) or range, while categorical variables were expressed as number of cases (n) and percentage (%). For the four children with sequenced isolates, nSOFA scores and other clinical features were presented as descriptive clinical metadata alongside bacterial virulence gene profiles using tabular and visualization methods. No inferential statistical tests, multivariate modeling, or genotype–phenotype correlation analyses were performed.

## Results

### Clinical characteristics of the 4 neonates

This study included four newborns diagnosed with *L. monocytogenes* sepsis confirmed by blood culture, with gestational ages ranging from 32 to 38 weeks. Three were preterm infants (75%, cases 1, 2, and 3), and one was a full-term infant (case 4). Birth weight distribution was relatively dispersed, with most being of average weight. All four cases were early-onset sepsis, with onset ranging from 1 to 48 h after birth, with three cases developing symptoms within 24 h of birth. Placental pathology revealed that the mothers of two infants (case 1 and case 3) had chorioamnionitis. All infants required respiratory support; three received invasive mechanical ventilation, and one required non-invasive ventilation.

Clinically, all four neonates presented with poor responsiveness and dyspnea at onset, suggesting systemic infection with respiratory involvement, and three also developed fever. Early laboratory findings showed elevated inflammatory markers or thrombocytopenia in all four cases, further supporting the diagnosis of sepsis. Sepsis-related multisystem involvement was also observed: one neonate developed purpura, and two developed significant jaundice. Regarding organ dysfunction, two neonates presented with tachycardia and hypotension, suggesting circulatory instability. Liver involvement was evident in Case 2, with marked elevation of transaminases, while Cases 1 and 4 showed varying degrees of coagulation abnormalities; Case 4 also had marked hyperbilirubinemia and bleeding tendency. No definite liver injury was observed in Case 3. In terms of renal involvement, Case 2 had oliguria, suggesting possible renal hypoperfusion, whereas the remaining cases had no documented oliguria or acute kidney injury. Physical examination did not record hepatosplenomegaly in any of the four neonates.

Case 1 received meropenem for 15 days, then switched to piperacillin/tazobactam for 4 days, for a total treatment duration of 19 days; Case 2 received meropenem for 21 days; Case 3 received meropenem for 13 days; Case 4 received a total treatment duration of 27 days, initially using penicillin combined with ceftazidime, followed by amoxicillin/clavulanate potassium combined with ceftazidime, and later switched to meropenem combined with vancomycin.

Severe complications primarily affected the nervous and circulatory systems: Case 1 progressed to septic shock, with purulent meningitis and intracranial hemorrhage; Case 2 also progressed to septic shock, but without clear central nervous system hemorrhage; Cases 3 and 4 did not develop clear shock or central nervous system complications, and their overall condition was relatively mild. All four children survived and were discharged after comprehensive treatment, with a mortality rate of 0% (Table 1).

**Table 1:** Clinical characteristics and outcomes of neonates with *Listeria monocytogenes* (*n =* 4).

Case	Gestational age (w)	Birth weight (g)	Onset of illness (h)	Chorioamnionitis	Mode of delivery	Mechanical ventilation	Septic shock	Purulent meningitis	Intracranial hemorrhage	Length of stay (d)	nSOFA score
Case1	32	1780	1	Confirmed	Cesarean section	Yes	Yes	Yes	Yes	34	4
Case2	36	3,280	4	Suspected	Cesarean section	Yes	Yes	No	No	21	7
Case3	35	2,940	24	Confirmed	Cesarean section	Yes	No	No	No	13	2
Case4	38	3,380	48	Not submitted for testing	Vaginal delivery	No	No	No	No	27	2

All four children survived. Case 1 showed slightly delayed development during early follow-up and received rehabilitation treatment; at the 9-month and 15-day follow-up, the developmental assessment was moderate. Case 2 was followed up to 5 years old and recorded neurodevelopmental delay, but no hearing impairment, epilepsy/seizures, hydrocephalus, encephalomalacia, ventricular enlargement, motor disorders, or recurrent infections. Case 3 was followed up to 4 years old, and no obvious neurological or growth and developmental abnormalities were observed. Case 4 was followed up for 2 months and no obvious sequelae were observed. No hearing impairment, epilepsy/seizures, hydrocephalus/encephalomalacia/ventricular enlargement, motor disorders, or recurrent infections were recorded in any of the four cases.

### WGS and virulence gene profiles of 4 isolates

Whole-genome sequencing was performed on the *L. monocytogenes* strains isolated from the blood cultures of four children, and the carriage of virulence factors was analyzed. MLST typing of four *L. monocytogenes* isolates showed that: Case 1 and Case 2 strains were both ST1, belonging to CC1, lineage I; Case 3 strain was ST155, belonging to CC155, lineage II; Case 4 strain was ST121, belonging to clonal complex CC121, lineage II. These results suggest that neonatal *L. monocytogenes* sepsis in this group was caused by strains with different sequence types and clonal complexes.

### Unique virulence gene patterns by case

**Figure 2:**
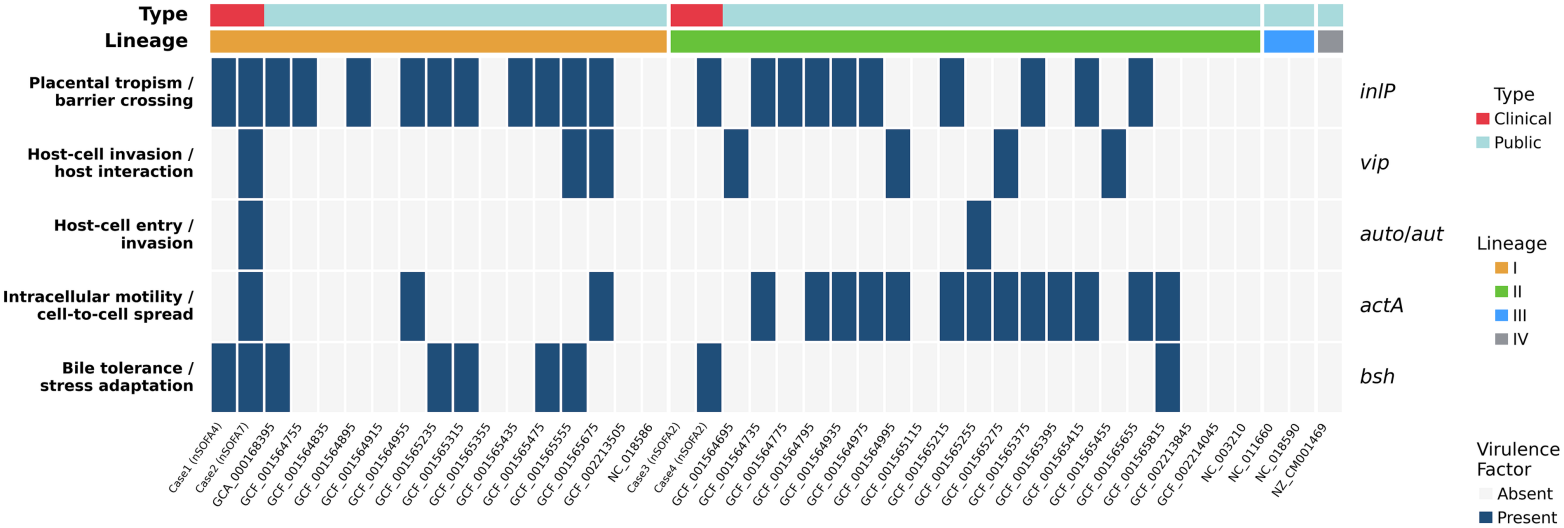
Heatmap of selected accessory virulence genes in neonatal *Listeria monocytogenes* isolates and public reference genomes. Presence/absence profiles of five selected accessory virulence genes, including *inlP, vip, auto/aut, actA, and bsh*, were analyzed in the four neonatal clinical isolates from this multicenter study and 39 public *L. monocytogenes* reference genomes used in the phylogenetic analysis. Isolates are arranged according to MLST lineage, with lineage I and lineage II genomes grouped separately, and lineage III/IV genomes placed at the right side of the heatmap. The upper annotation bars indicate isolate type and MLST lineage. Solid blue indicates gene presence, and light gray indicates gene absence. Functional annotations on the left summarize the reported roles of these genes, including placental tropism and barrier crossing (InlP), host-cell invasion or host interaction (Vip and Auto/aut), intracellular motility and cell-to-cell spread (ActA), and bile tolerance or stress adaptation (Bsh). The numbers in parentheses following the four clinical case labels represent the nSOFA score as descriptive clinical information and do not represent statistical correlation.

To determine whether the observed accessory virulence gene profiles reflect isolate-specific variation or phylogenetic background, a total of 43 public reference genomes were used in phylogenetic analysis. The same VFDB-based workflow and screen criteria were applied to the four neonatal isolates and the public reference genomes, and the presence or absence profiles of *actA, auto, vip, bsh,* and *inlP* were visualized in a heatmap (Figure 2).

The nSOFA scores for Cases 1–4 were 4, 7, 2, and 2, respectively. The isolate from Case 2, which had the highest nSOFA score, carried all five selected auxiliary virulence genes covering various functional categories, including placental tropism and barrier crossing (*inlP*), host-cell entry, invasion, and interaction (*vip* and *auto*), intracellular motility and cell-to-cell spread (*actA*), and bile tolerance or gastrointestinal stress survival (*bsh*). The isolate from Case 1 harbored inlP and bsh. In contrast, the isolates from Cases 3 lacked all five selected auxiliary virulence genes, whereas the isolate from Case 4 lacked *actA, auto* and *vip.*

Among the 39 lineage I/II genomes, *actA* carriage showed a significant association with lineage II background by Pearson’s *χ*^2^ test (*p* = 0.001), whereas *bsh* carriage showed a significant association with lineage I background by Fisher’s exact test (*p* = 0.026).

### Phylogenetic clustering based on core-genome SNPs

**Figure 3:**
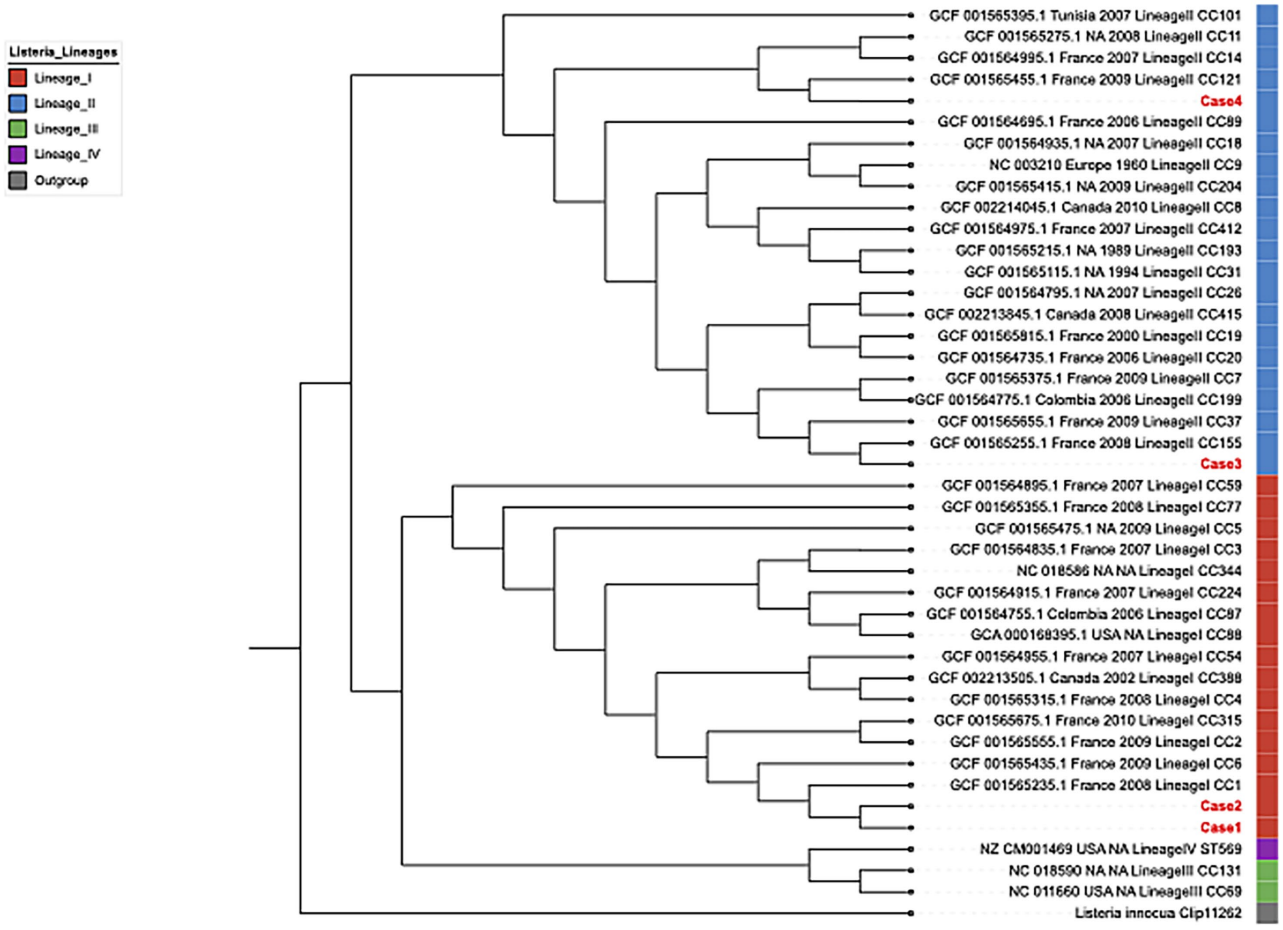
Core genome phylogenetic alignment of four neonatal *L. monocytogenes* isolates. The phylogenetic tree shows the clustering of each isolate with the representative strain of the known clonal complex (CC). Case 4 clusters with the representative strain of CC121 (GCF 001565455.1 France 2009), and case 3 clusters with the representative strain of CC155 (GCF_001565255.1, France, 2008). Cases 1 and 2 form a tightly linked pair, closely related to the representative strain of CC1 (GCF_001565235.1, France, 2008).

Phylogenetic reconstruction demonstrated that all four clinical isolates were stably positioned within their respective lineage branches, showing high genetic proximity to representative strains from specific CCs available in public databases. Specifically, Case 4 was most closely related to the CC121 representative strain (GCF 001565455.1 France 2009), while Case 3 exhibited the closest phylogenetic affinity with the CC155 representative strain (GCF_001565255.1, France 2008). Notably, Cases 1 and 2 formed a distinct, tightly linked cluster on the phylogenetic tree, demonstrating high similarity to the CC1 representative strain (GCF_001565235.1, France 2008) (Figure 3).

## Discussion

In this case series of neonatal *L. monocytogenes* sepsis with matched isolate WGS, all infections were early onset, yet clinical severity varied substantially (cases 1–2 more severe than cases 3–4). Across isolates, core virulence determinants implicated in adhesion, invasion, motility, and stress survival were conserved, while some auxiliary virulence genes exhibited distinct presence/absence patterns. Because only four clinical isolates were sequenced, the observed case-level differences in virulence gene carriage should be regarded as descriptive observations. These findings may help generate testable hypotheses regarding strain-level genomic diversity in neonatal *L. monocytogenes* sepsis, but they do not establish a relationship with clinical phenotype heterogeneity (Table 2).

**Table 2:** Whole genome sequencing was performed on *Listeria monocytogenes* isolates (*n =* 4).

Isolate	ST	CC	Lineage	Molecular serogroup	Average depth	N50	Contigs	GC%	Raw reads	Clean reads
Case1	1	CC1	I	IVb	146	51,806	118	39.8	3,546,364	3,202,076
Case2	1	CC1	I	IVb	287	72,504	83	39.8	6,955,040	6,300,148
Case3	155	CC155	II	IIa	201	109,332	49	39.5	4,809,104	4,514,914
Case4	121	CC121	II	IIa	350	236,590	50	39.2	7,903,458	7,542,568

Previous studies have suggested that the overall incidence of neonatal listeriosis is low but clinically severe. *L. monocytogenes* accounted for 4.7% of positive cultures for neonatal sepsis in the Paris region from 2019 to 2021 (Sikias et al., 2023). Our study observed a 3.7% rate, along with reports of 2–3% in other regions (Qu et al., 2022; Paraparambil Vellamgot et al., 2025), suggesting that *L. monocytogenes* is a rare cause of culture-confirmed neonatal sepsis. However, continued clinical surveillance is necessary because neonatal listeriosis can lead to serious perinatal complications, including early-onset sepsis, meningitis, septic shock, and death (Disson et al., 2025). In this context, the local proportions observed in our cohort provide regional clinical evidence.

Based on previous mechanistic studies, some of the gene-level observations in this series are biologically plausible. Firstly, Faralla et al. showed that the secreted InlP targets host cytoplasmic protein afadin, enhancing the transcytosis across epithelial barriers, thereby promoting placental infectivity (Faralla et al., 2018). In animal models, loss of *inlP* reduces placental colonization and attenuates fetal infection risk (Faralla et al., 2018). In our report, only the isolate from Case 3 (nSOFA score = 2) lacked *inlP*. While such a pattern is compatible with reduced barrier crossing and/or attenuated placental tropism, functional validation would be required to determine whether *inlP* status materially influences neonatal disease severity in this setting. Secondly, ActA mediates actin-based motility and cell-to-cell spread in *L. monocytogenes* (Czuczman et al., 2014) and contributes to intracellular dissemination through actin “comet tail” formation (Kuhn and Enninga, 2020). ActA has also been linked to avoidance of host intracellular clearance mechanisms, including autophagy-related pathways (Pillich et al., 2017). In the present study, ActA was detected only in the isolate from Case 2, the most severe presentation (septic shock with meningitis and intracranial hemorrhage). This observation is consistent with the hypotheses that ActA-associated intracellular spread and immune evasion may facilitate broader dissemination and a heightened systemic inflammatory response. Thirdly, of the four blood culture isolates, the *auto* and *vip* genes were present only in isolates Cases 2, while this combination was absent in the other three isolates. Previous studies have shown that Auto (an autolysin) is involved in non-phagocytic cell invasion, and its deletion mutants exhibit attenuated virulence in animal models (Cabanes et al., 2004). Vip is a cell wall anchoring factor displayed on the bacterial surface via type A sorting enzyme (SrtA). It has been reported to bind to the host receptor Gp96, thereby promoting bacterial entry into the cell and the later stages of infection (Cabanes et al., 2005). Last, *L. monocytogenes* must tolerate the antimicrobial effects of bile to establish infection and colonize the human gastrointestinal tract. Bile hydrolysis assays showed that *L. monocytogenes* contains a bile salt hydrolase gene (*bsh*). Transcriptional and enzyme activity data further indicated that σB is a key regulator of *bsh* expression. In addition to loss of bile hydrolysis activity, σB-deficient mutants also exhibited significant bile salt hypersensitivity (Begley et al., 2005).

The outcome of neonatal *L. monocytogenes* infection is influenced by factors such as gestational age, birth weight, immune maturity, maternal infection status, placental inflammation, and time of onset. Prematurity, exposure to chorioamnionitis, and severe early postnatal infections can increase the risk of severe illness (Disson et al., 2025). In the present case series, all four patients had broadly similar high-risk perinatal backgrounds, yet their trajectories diverged. Therefore, in addition to the established effects of host and treatment-related factors of disease severity, we propose a new explanatory dimension: differences in strain virulence. In the present study, the most severe Case 2 simultaneously carried five virulence genes, covering the functions including placental tropism and barrier crossing, host-cell entry, invasion, and interaction, intracellular motility and cell-to-cell spread, and bile tolerance or gastrointestinal stress survival. While the isolate from Case 3 lacked all five of these auxiliary virulence genes. Furthermore, Cases 1 and 2 belong to the ST1/CC1/lineage I group, while Cases 3 and 4 belong to the lineage II-related clonal complex. Previous large-scale comparative genomics studies have shown differences in clonal complex distribution and virulence between *L. monocytogenes* isolates from food and clinical sources (Maury et al., 2016). Maury et al. (2016) analyzed over 6,000 food and clinical isolates, classifying some clones as food-associated clones, such as CC9 and CC121; others as infection-associated clones, such as CC1 and CC2. In our cases, Cases 1 and 2 belonged to CC1/lineage I, while Case 3 belonged to CC155/lineage II, and Case 4 belonged to CC121/lineage II, indicating that the different case strains were in different clonal contexts.

In our expanded comparative analysis, *actA* showed a lineage II-associated distribution, whereas *bsh* showed a lineage I-associated distribution. Functionally, ActA is involved in actin-based intracellular motility and cell-to-cell spread, whereas Bsh contributes to bile tolerance and gastrointestinal stress adaptation. Previous comparative genomics studies have shown that the virulence of *L. monocytogenes* is closely associated with evolutionary lineage and MLST clonal complex background (Disson et al., 2021). Highly virulent clonal complexes (CCs) and major virulence islands (such as LIPI-3 and LIPI-4) are not randomly distributed but enriched in specific lineages or CCs (Lakicevic et al., 2021). The lineage-associated distribution may reflect different ecological or infection-stage adaptations among *L. monocytogenes* lineages. However, gene presence alone should not be equated with virulence strength, and these results should not be interpreted as evidence that *actA* or *bsh* determines clinical severity. The clinical interpretation of WGS-derived virulence profiles in neonatal *L. monocytogenes* sepsis should account for MLST lineage and CC background, because selected virulence genes may be lineage-structured rather than purely isolate-specific.

## Conclusion

These findings highlight strain-level differences in nSOFA scores and accessory virulence gene carriage. However, these findings should be interpreted within a phylogenetic context and cannot directly establish genotype–phenotype relationships. Except for the resistance analysis, WGS may have more value to generate testable hypotheses in rare but high-risk neonatal infections by characterizing virulence lineages. Larger-scale, phylogenetically matched cohorts, combined with standardized clinical data and functional validation, are needed before incorporating virulence gene profiles into risk stratification or follow-up strategies.

## Limitations

This study should be considered a descriptive and hypotheses-generating analysis. The limited number of isolates available for WGS restricts formal statistical inference and the general applicability of the results. Potential confounding factors, including gestational age, birth weight, maternal comorbidities, perinatal factors, nSOFA score timing, and specific treatment strategies at each hospital, could not be fully controlled for. Third, we implemented a highly conservative curation strategy where each background MLST or CC was represented by only a single public reference genome to prevent tree-skewing biases. While this effectively established an un-biased macro-evolutionary context, it inherently limited our resolution to capture fine-grained intra-CC genetic diversity or micro-scale local geographic clustering. Consequently, the evolutionary distances between our clinical isolates and contemporary regional strains might be artificially highlighted. Furthermore, virulence gene analysis was based on database-dependent annotation, relying solely on presence/deletion profiles without transcriptomic, proteomic, *in vitro*, or *in vivo* functional validation. Larger-scale prospective neonatal cohort studies, incorporating standardized clinical data, more sequenced isolates, and functional studies are needed to validate these exploratory findings.

## Data Availability

The original contributions presented in the study are publicly available. This data can be found here: https://doi.org/10.6084/m9.figshare.32825225.

## Glossary

ActA: Actin assembly-inducing protein
Auto: Autolysin
BSH/bsh: Bile salt hydrolase
CC: Clonal complex
CGE: Center for Genomic Epidemiology
ClpC/ClpE: Caseinolytic protease chaperone proteins
CRP: C-reactive protein
DIC: Disseminated intravascular coagulation
ER: Endoplasmic Reticulum
FbpA: Fibronectin-binding protein A
Gp96: Glycoprotein 96
InlP: Internalin P
iTOL: Interactive Tree Of Life
Lap: Listeria adhesion proteins
MLST: Multilocus sequence typing
N50: N50 statistic of genome assembly contiguity
NGS: Next-generation sequencing
nSOFA: Neonatal Sequential Organ Failure Assessment
ORF: Open reading frame
PCT: Procalcitonin
PE150: Paired-end 150-bp sequencing
σB: Sigma B
ST: Sequence type
VFDB: Virulence Factor Database
Vip: Virulence protein
WGS: Whole-genome sequencing
